# 3D Printed Biomimetic Rabbit Airway Simulation Model for Nasotracheal Intubation Training

**DOI:** 10.3389/fvets.2020.587524

**Published:** 2020-11-27

**Authors:** Gunpreet Oberoi, M. C. Eberspächer-Schweda, Sepideh Hatamikia, Markus Königshofer, Doris Baumgartner, Anne-Margarethe Kramer, Peter Schaffarich, Hermann Agis, Francesco Moscato, Ewald Unger

**Affiliations:** ^1^Center for Medical Physics and Biomedical Engineering, Medical University of Vienna, Vienna, Austria; ^2^Department of Conservative Dentistry and Periodontology, School of Dentistry, Medical University of Vienna, Vienna, Austria; ^3^Department/Hospital for Companion Animals and Horses, University of Veterinary Medicine, Vienna, Austria; ^4^Austrian Center for Medical Innovation and Technology, Wiener Neustadt, Austria; ^5^Center for Biomedical Research, Medical University of Vienna, Vienna, Austria; ^6^Ludwig Boltzmann Institute for Cardiovascular Research, Vienna, Austria; ^7^Austrian Cluster for Tissue Regeneration, Vienna, Austria

**Keywords:** additive manufacturing, animal trials, nasotracheal fiberoptic intubation, simulation model, medical imaging, support material removal, skills training

## Abstract

Rabbit inhalation anesthesia by endotracheal intubation involves a higher risk among small animals owing to several anatomical and physiological features, which is pathognomonic to this species of lagomorphs. Rabbit-specific airway devices have been designed to prevent misguided intubation attempts. However, it is believed that expert anesthetic training could be a boon in limiting the aftermaths of this procedure. Our research is aimed to develop a novel biomimetic 3D printed rabbit airway model with representative biomechanical material behavior and radiodensity. Imaging data were collected for two sacrificed rabbit heads using micro-computed tomography (μCT) and micro-magnetic resonance imaging for the first head and cone beam computed tomography (CBCT) for the second head. Imaging-based life-size musculoskeletal airway models were printed using polyjet technology with a combination of hard and soft materials in replicates of three. The models were evaluated quantitatively for dimensional accuracy and radiodensity and qualitatively using digital microscopy and endoscopy for technical, tactic, and visual realism. The results displayed that simulation models printed with polyjet technology have an overall surface representation of 93% for μCT-based images and 97% for CBCT-based images within a range of 0.0–2.5 mm, with μCT showing a more detailed reproduction of the nasotracheal anatomy. Dimensional discrepancies can be caused due to inadequate support material removal and due to the limited reconstruction of microstructures from the imaging on the 3D printed model. The model showed a significant difference in radiodensities in hard and soft tissue regions. Endoscopic evaluation provided good visual and tactile feedback, comparable to the real animal. Overall, the model, being a practical low-cost simulator, comprehensively accelerates the learning curve of veterinary nasotracheal intubation and paves the way for 3D simulation-based image-guided interventional procedures.

## Introduction

Procuring small and large animal specimens is imperative for anatomical understanding (1). Thorough knowledge of spatiotemporal skeletal anatomy is applied by biomedical engineers and researchers in various disciplines to develop neuromuscular devices and prostheses (2). It is also useful for developing new surgical protocols and implants (3). Nevertheless, as part of good scientific practice, one must endeavor to employ minimal animal specimens for training and investigation purposes (4).

Currently available models for skills training in veterinary medicine include animal specimens, simulation models, and virtual reality (5–7). Use of animal dissection surges expenses, requires special preparation, causes toxicity due to formalin, might not be reusable, and most importantly requires animal sacrifice, increasing the load on animal experiments and ethical approvals (8–12). Virtual reality might be excessively expensive for budding surgeons and not universally available (5, 13–15). Employing an additively manufactured animal model prior to the final animal surgery is proposed to relieve this burden.

In pre-clinical experiments with small animals, rabbits are very useful in studies concerning sinusitis, oral and maxillofacial surgeries, and middle ear defects, owing to their close resemblance to humans (16, 17). Studies employing surgical procedures require rabbit anesthetic intubation (18). Rabbit endotracheal intubation is one of the most critical among various small animals due to its narrow oral passage, large tongue and torus as well as a long epiglottis (19). The oro-pharynx is traumatized by repeated tube insertion, and it is believed that rigorous anesthetic training can help improve the prognosis of this procedure. Clinical perceptions of the intubated creatures recommend that nasotracheal intubation can be utilized as an alternative, atraumatic elective strategy for a protected and compelling rabbit intubation (9, 20, 21).

To gain technical skills, additive manufacturing (AM) of anatomical simulation models based on computed tomography (CT) and magnetic resonance imaging (MRI) is an extremely powerful tool in reducing the load on animals (22). Physical models can enhance the learning curve of the surgeons, especially in challenging anatomical conditions. Attempts have been made to fabricate human simulation models using multi-material additive manufacturing technology. However, recreating animal anatomy in this magnitude is yet unexplored (13, 23–26). The existing AM animal simulation models lack the realistic complex nasopharyngeal anatomy and only represent a part of the skull (27). Hence, complex maneuvers like nasotracheal intubation, nasal endoscopy, and polypectomy are impossible to perform, ultimately leading to animal sacrifice.

The ideal reenactment model would closely resemble animal tissue both geometrically and haptically, be reproducible, and be sensibly evaluated. A measured framework, taking into consideration a cost-effective, replaceable supplement, would offer a perfect answer for preparing veterinary residents in managing complex and routine nasopharyngeal procedures. Hence, in this study, we used the printer based on polyjet technology, which provided the flexibility of selection over a wide range of materials differing in hardness, color, and other physical characteristics, befitting the specific structures (28–30). The resolution is as fine as 600 dots per inch (dpi) with 42.3 μm in the XY plane and 16–30 μm in the Z direction (layer thickness), making it the printer of choice for replicating intricate nasal geometry (4, 31, 32).

Equally, the bedrock of superior printing lies in acquisition of data through digital and radiological three-dimensional imaging techniques (33). We employed micro-magnetic resonance (μMRI), micro-computed tomography (μCT), and cone beam computed tomography (CBCT) for imaging pre-sacrificed rabbit heads to obtain the three-dimensional data set in high resolution. The data were then rendered and segmented into printable Standard Tessellation Language (STL) file using Materialize Mimics 21.0 computer software. A life-size multi-material rabbit nasotracheal simulation model was constructed and compared both quantitatively and qualitatively with the real animal specimen.

Our prototype biomimetic nasotracheal simulation model has the potential in embracing the 3Rs (reduction, refinement, and replacement) of animal experiments, providing a true-to-life model for hands-on training for several surgical and radiographic diagnostic procedures and medical device development, which otherwise are only possible to perform in live animals. With advanced utilization of AM, the ethical, financial, and humane issues raised by the procurement of live animals for skill development can be decreased.

## Materials and Methods

### Animal Specimens

We received two sacrificed female ram rabbit specimens, weighing ~3.5 kg each. Ethics permission was not required for our study since we used sacrificed animals from an ongoing study in the Department of Biomedical Research, Medical University of Vienna, Austria, and the Department/Hospital for Companion Animals and Horses, University of Veterinary Medicine, Vienna, Austria. The ethical permission for their ongoing studies was already acquired by the Ethic Commission of the University of Vienna. Those cadavers were chosen because of the representative weight utilized for animal laboratory experiments for human research (3.0–5.5 kg) as well as their bigger airway diameter to facilitate nasotracheal intubation with the smallest 2.0 uncuffed endotracheal tube. It was proposed that smaller-sized rabbits under 1.5 kg of weight are unable to be intubated nasotracheal with even the smallest-sized endotracheal tube. The oral and the nasal cavities of the specimens were carefully swiped for removal of saliva, and then an inflatable balloon was inserted to separate the tongue from the hard palate in order to get a better radiographic view of the airway. The animal specimens were named as 1 and 2 during the course of the study.

### Image Acquisition

**Figure 1:**
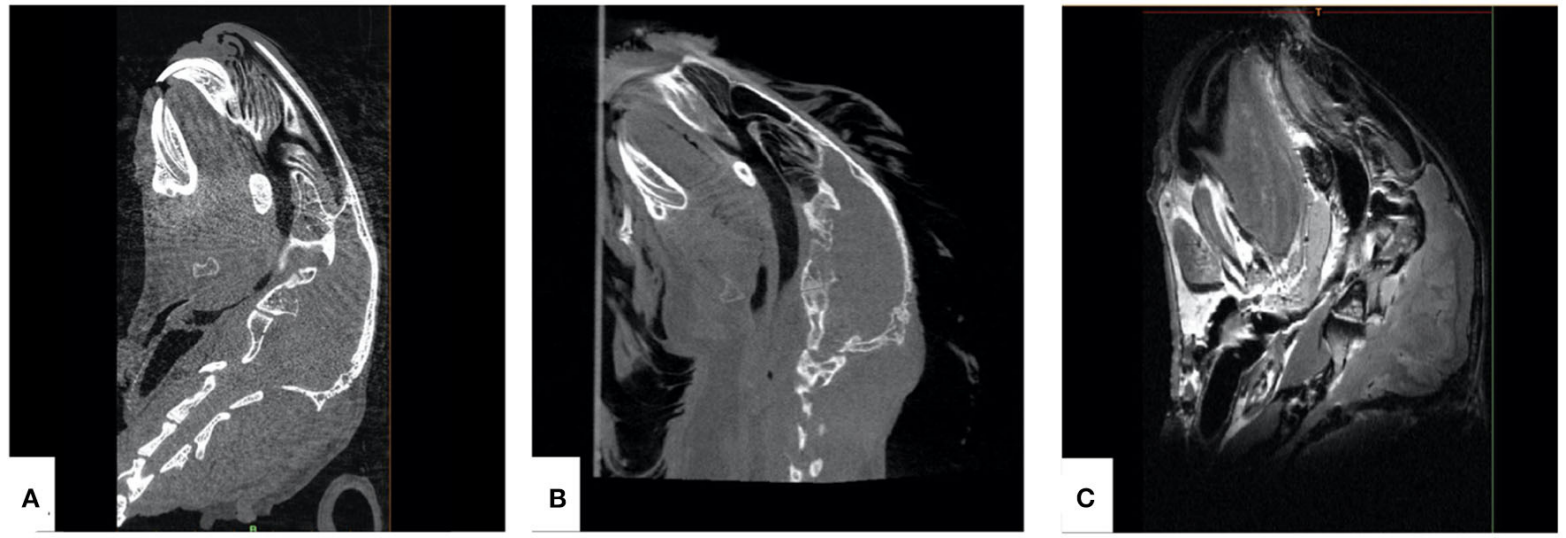
Sagittal sections from multiple imaging techniques used for the acquisition of data for additively manufactured rabbit airway models. **(A)** μCT, **(B)** cone-beam computed tomography, and **(C)** μMRI.

Multimodal image acquisition was performed for specimen 1 using μCT of Siemens Inveon® μCT (Siemens Medical Solutions, Inc. Molecular Imaging, Knoxville, United States), with the following parameters: 80 kV peak voltage, 200 ms exposure time, 200 mA tube current, 97 μm voxel size, and 0.1 mm slice thickness (Figure 1A), and two-dimensional μMRI (BioSpec® 94/30USR MRI, Bruker, Bruker Medical, Ettlingen, Germany), with the following parameters: 9.40 T magnetic field strength, 23.06 ms echo time, 400 Hz frequency, 439 μm pixel size, and 1.4 mm slice thickness (Figure 1C). CBCT (Planmed Verity®, Planmeca Oy, Helsinki, Finland) was performed for specimen 2 using the following parameters: tube voltage 80–90 kVp, tube current 3.2–12 mA, slice thickness 0.2 mm, voxel size 0.2 mm, detector resolution 1,575 × 1,969 pixel, image resolution 0.2000 × 0.2000 pixel spacing, and field of view 130 × 160 mm (Figure 1B). μMRI for specimen 1 was performed to get a detailed information regarding the spatio-temporal arrangement of the soft tissue anatomy in the nasopharyngeal airway, although it was not translated for 3D printing.

### 3D Model Generation

**Figure 2:**
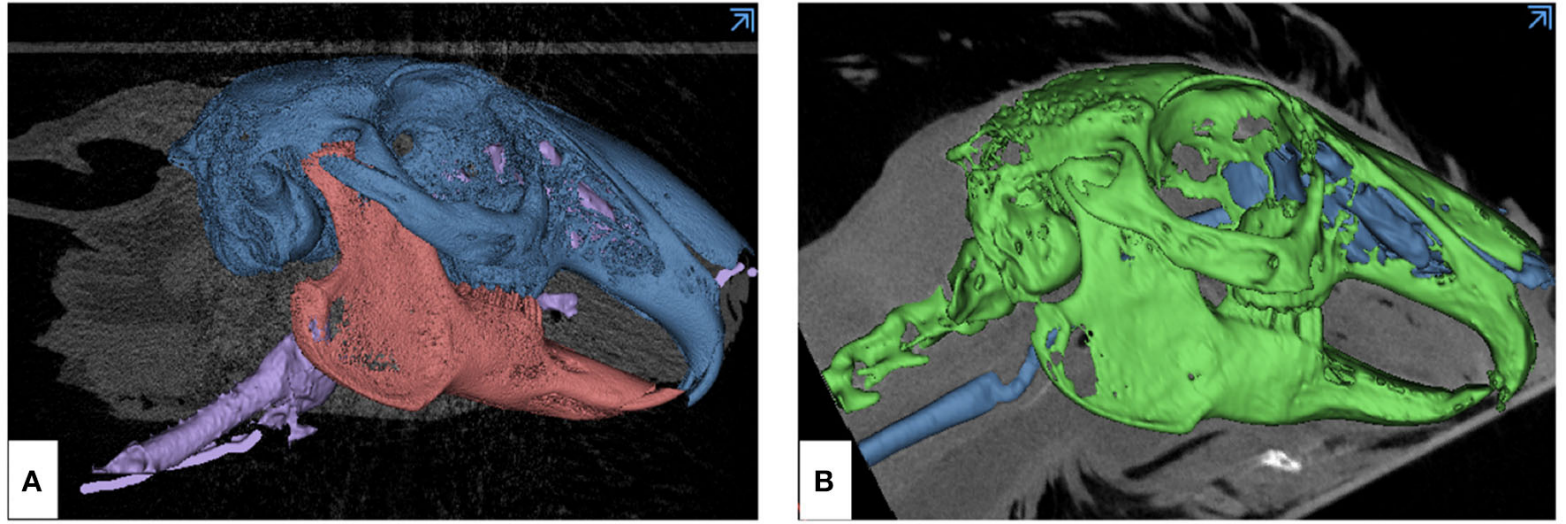
Overview of the skeletal and mucosal masks created from DICOM imaging data of animal models using Materialize Mimics Research 21.0 segmentation software for translating the information into STL format. **(A)** μCT-derived mask: blue—skull with maxilla, pink—mandible, violet—nasotracheal airway, and **(B)** CBCT-derived mask: green—skull with maxilla and mandible, blue—nasotracheal airway.

Imaging data obtained as Digital Imaging and Communication in Medicine (DICOM) files from both imaging modalities, μCT and CBCT, were rendered and segmented using Materialize Mimics Research 21.0 software (Materialize, Leuven, Belgium). Anatomical/missing parts were post-processed using Materialize 3-Matic 13.0 software (Materialize, Leuven, Belgium) (Figure 2). Post-segmentation processing consisted of spike reduction, adaptive remesh, and smoothening functions. Spike reduction removed spike-shaped noise from the surface, with the smallest spike size chosen as 0.10 mm and accuracy of surface preservation as 0.1000 mm. During spike reduction, we chose to preserve the surface structure and reduce the triangles to make processing of larger and complex areas possible. Adaptive remesh was performed to reduce image size and speed up the model designing process. This function allows to remesh the entity with a finer control while preserving the geometry. The parameters selected for adaptive remesh were 0.1000 mm as shape quality threshold, 0.0300 mm as maximum geometrical error, minimum and maximum triangle edge length of the resulting mesh as 0.0500 and 0.1000, respectively, number of iterations as 10 to meet the shape quality threshold, and growth rate of 25% to allow triangles 1 mm greater from the surface to have 25% more than the maximum triangle edge length. Following this, STL (or STereoLithographic) files were created from both specimens to translate the imaging information into a printable format using Materialize 3-Matic 13.0 software.

### 3D Printing of Rabbit Head Model

The STL file created consequently was used to additively manufacture a 1:1 scaled model of the rabbit head with PolyJet^TM^ printer Connex3 Objet500 (Stratasys, EdenPrairie, MN, United States of America). The skeletal part of the model was printed in Vero Pure White mixed with TangoPlus. After reviewing the biomechanical properties of multiple materials, we found that Vero Pure White RGD835 (Stratasys Ltd., Eden Prairie, MN), a rigid white opaque material, would be suitable for bone. The material used to replicate the skin and mucosa had to be elastic and soft. TangoPlus FLX930 (Stratasys) is a rubberlike material that resembles the biomechanical properties of mucosa and skin. The material selection was based on the biomechanical tests performed by our research team. Test samples in various rigid and flexible 3D printed materials available for polyjet printing were characterized in three build orientations, namely, XY, YZ, and ZX. DIN EN ISO-527-1/2-based tensile tests and shore hardness were performed to analyze the modulus of elasticity and hardness of these materials and compare it to those of biological hard and soft tissues to improve their application in simulation models (34, 35). The printing time of the models was 60 min, and it was possible to print all the models in one cycle. The models were then cleaned manually for gross support material (SUP706) removal and later flushed carefully using waterjet. The models were then placed in 2% sodium hydroxide solution for 30 min and rinsed with water.

### Dimensional Accuracy Evaluation

The AM model was scanned using the CT of multimodality Siemens Inveon® μCT (Siemens Medical Solutions, Inc., Molecular Imaging, Knoxville, United States) for small animals using the following parameters: 60 kV peak voltage, 250 mA tube current, 200 ms exposure time, and 0.20 mm slice thickness. The DICOM data obtained from this scan was converted into an STL file using Materialize Mimics Research 21.0 software. The dimensional accuracy of the AM model was analyzed by point-based part comparison analysis between the custom-defined hard tissue anatomical landmarks on the STL file of the animal specimen and AM models using Materialize 3-Matic 13.0 software (Materialize, Leuven, Belgium). We choose the palatal cusp tips for all maxillary teeth, the posterior-most tip of the palatine bone for the Z–X plane orientation, the anterior-most tip of the nasal bone, the external occipital protuberance for the Y–Z plane orientation, and the midline of the incisors and the incisal edges for the X–Y plane orientation. The parts showing a mismatch of overlap were further segmented using segmentation analysis function and analyzed. The models underwent a second cycle of cleaning process as mentioned in “Section 3D model generation,” and dimensional evaluation using part-comparison analysis was repeated using the same protocol (36).

### Endoscopic Evaluation

**Figure 3:**
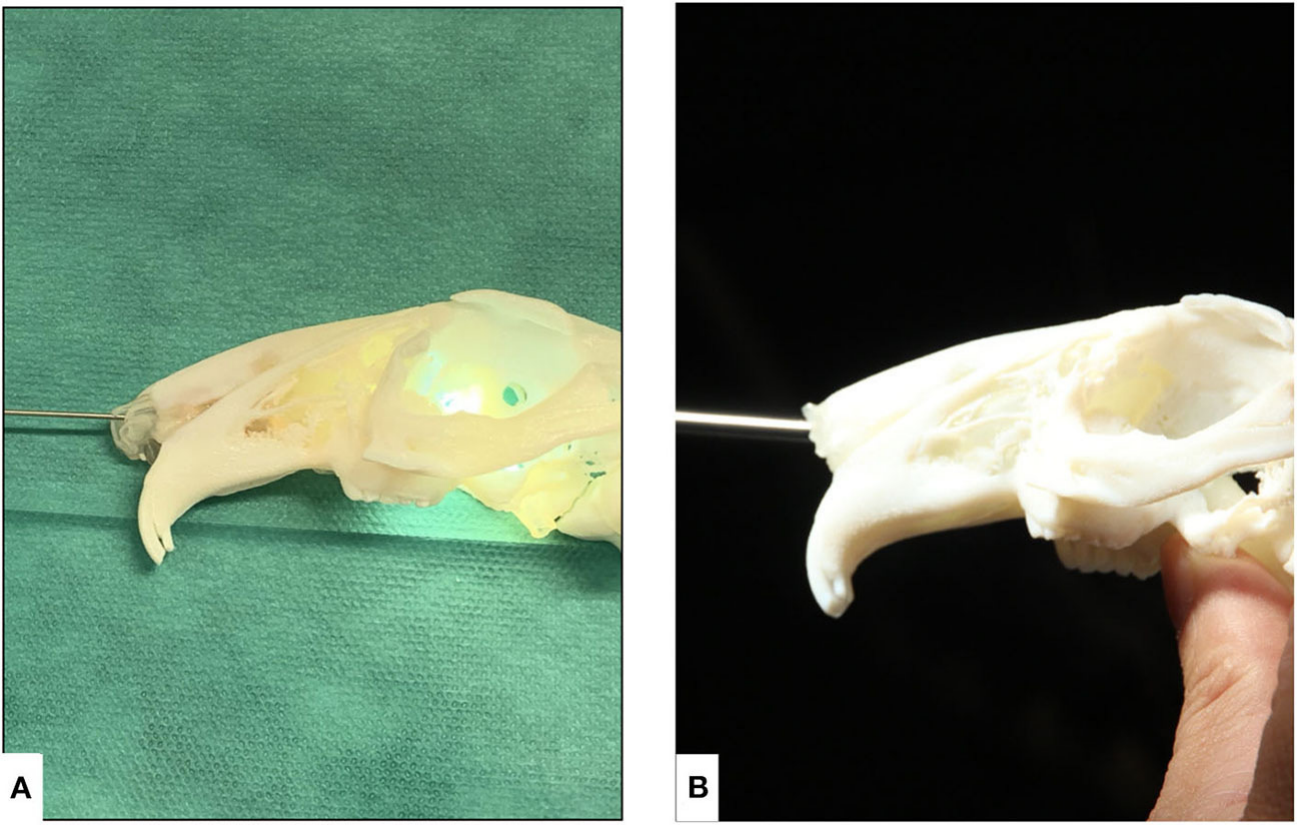
Endoscopic evaluation of nasotracheal airway in the additively manufactured rabbit airway models derived from μCT. **(A)** 1-mm rigid endoscope and **(B)** 2-mm rigid endoscope.

Both the animal specimen and the AM models were lubricated using 2% lidocaine-hydrochloride gel (Xylocain 2%-Gel Aspen Pharma Trading Ltd., Dublin, Ireland). Rigid video endoscopes at 1 and 2 mm (TELE PACK VET X LED, Karl Storz Se & Co. KG, Tuttlingen, Germany) were used for nasal intubation, and the anatomical details of the nasopharyngeal airway (retrograde endoscopy) along with the visual and tactile experience of the surgeon were recorded. The airway was constantly flushed in the AM models using tap water to increase patency and remove any remaining support material (Figure 3). The removal was confirmed with an endoscope by flushing with warm sterile saline (NaCl 0.9% B Braun, Meisungen) as under normal clinical conditions. After the complete removal of the supporting material, size 2 and size 2.5 uncuffed endotracheal tubes (RÜSCH Safety Clear® Germany) covered with 2% lidocaine-hydrochloride gel were used to successfully pass through the nasopharyngeal airway of the 3D printed model.

### Contamination Analysis Through Digital Microscopy

We interrogated both CBCT- and μCT-derived rabbit head models for surface contamination and the presence of support material using a digital microscope VHX-7000 (Keyence, Keyence International, Mechelen, Belgium). The VHX Series digital microscope has an increased depth of field and longer working distances than traditional optical microscopes, which also allows the generation of surface three-dimensional profiles to an accuracy of up to ±1 μm, depending on the magnification used. In terms of Keyence settings, the images were acquired using ×20 magnification, giving a field of view, for a single image, of 4,000 × 3,000 pixels. The sample was illuminated using a combination of direct and transmitted light. This combination of illumination had the net result of enhancing the contrast of the image.

### Model Radiodensity Evaluation

**Figure 4:**
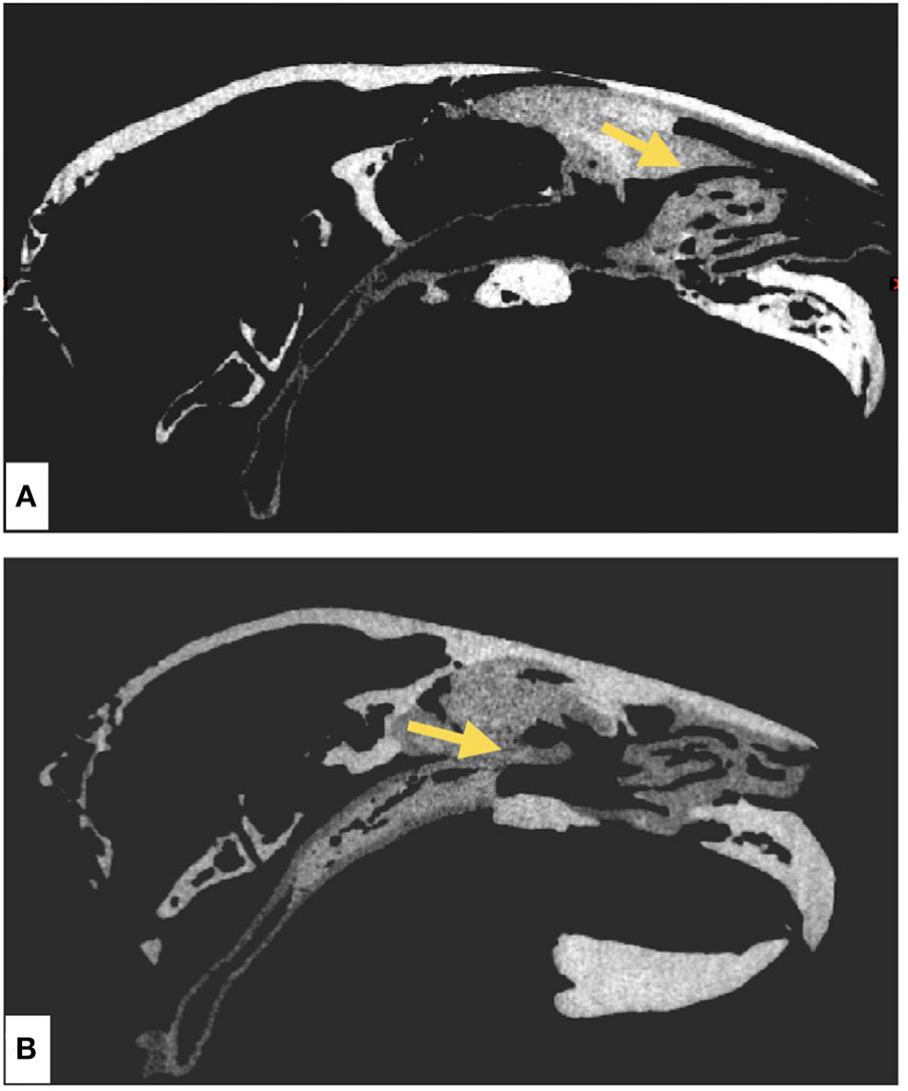
Radiodensity evaluation of skeletal and mucosal parts of the additively manufactured rabbit nasotracheal airway models using Analyze12.0 toolkit. The yellow arrow is a line profile that measures the gray value corresponding to the radiation attenuation of all points on the line. **(A)** Sagittal section of the CT scan of the μCT-derived model and **(B)** sagittal section of the CT scan of the CBCT-derived model.

For radiodensity evaluation of the models, we performed a CT scan to investigate the radiographic distinguishability between the mucosal and the bony areas, both qualitatively and quantitively, using the Hounsfield Unit (HU) analysis. A CT scan (SOMATOM Definition AS, Siemens Healthineers, Erlangen Germany) was employed in the study, with the following parameters: tube voltage 70 kVp, tube current time product 70 mAs, slice thickness 0.6 mm, voxel depth 0.6 mm, voxel height 0.1679 mm, and voxel width 0.1679 mm, including a total of 692 DICOM data sets, each with 512 × 512 pixel size, representing axial slices through the body. A comparable sagittal slice from both AM models was selected in Analyze 12.0 toolkit (AnalyzeDirect, Overland Park, United States of Kansas) to measure the HU related to the mucosal and the bony parts. The HU analysis was performed by taking line profiles at the defined regions of interest in mucosal and bony areas, respectively (Figure 4). The line profile measures radiation attenuation in terms of gray value and provides the mean, maximum, minimum, and standard deviation over all points related to the region of interest.

## Results

### Digital Model Design Was More Intricate for μCT-Derived Model

**Figure 5:**
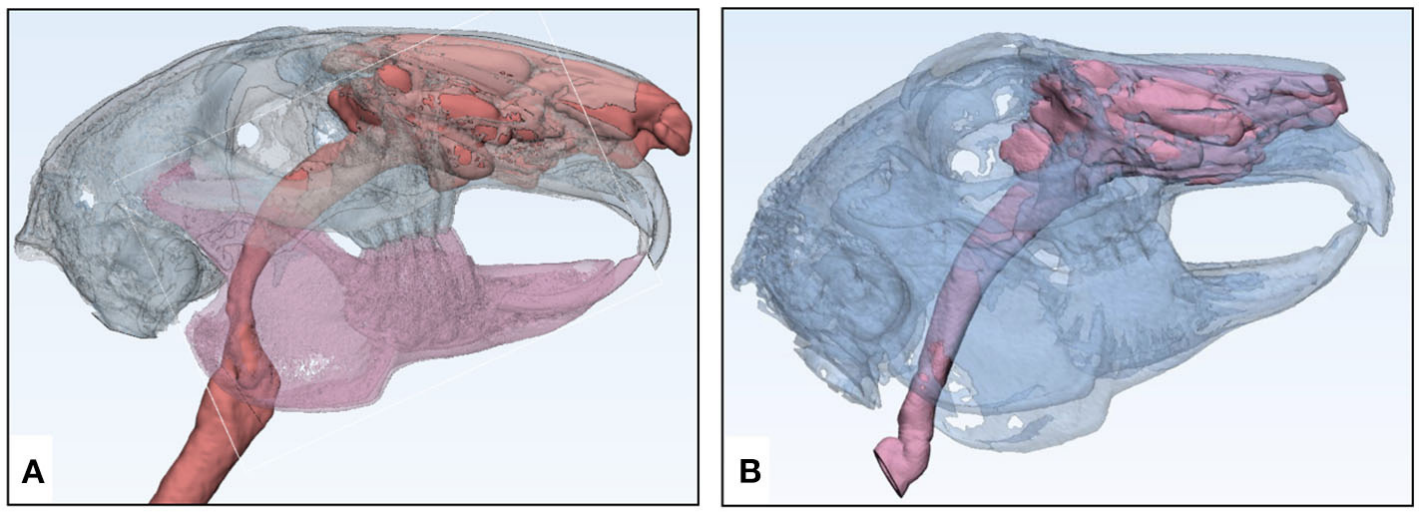
Digital model or STL files of imaging-based rabbit specimens with increased transparency created in Materialize 3-Matic 13.0 software for 3D printing of airway models. **(A)** μCT-based specimen STL and **(B)** CBCT-based specimen STL.

The rabbit specimen 1 underwent μCT and μMRI, while specimen 2 underwent CBCT. Digital models from both specimens were created using the same computer software, Materialize 3-Matic 13.0 (Figure 5). However, greater anatomical details were replicated in model 1 due to the higher resolution and better contrast in the former image acquisition modality.

### 3D Printed Rabbit Airway Model

**Figure 6:**
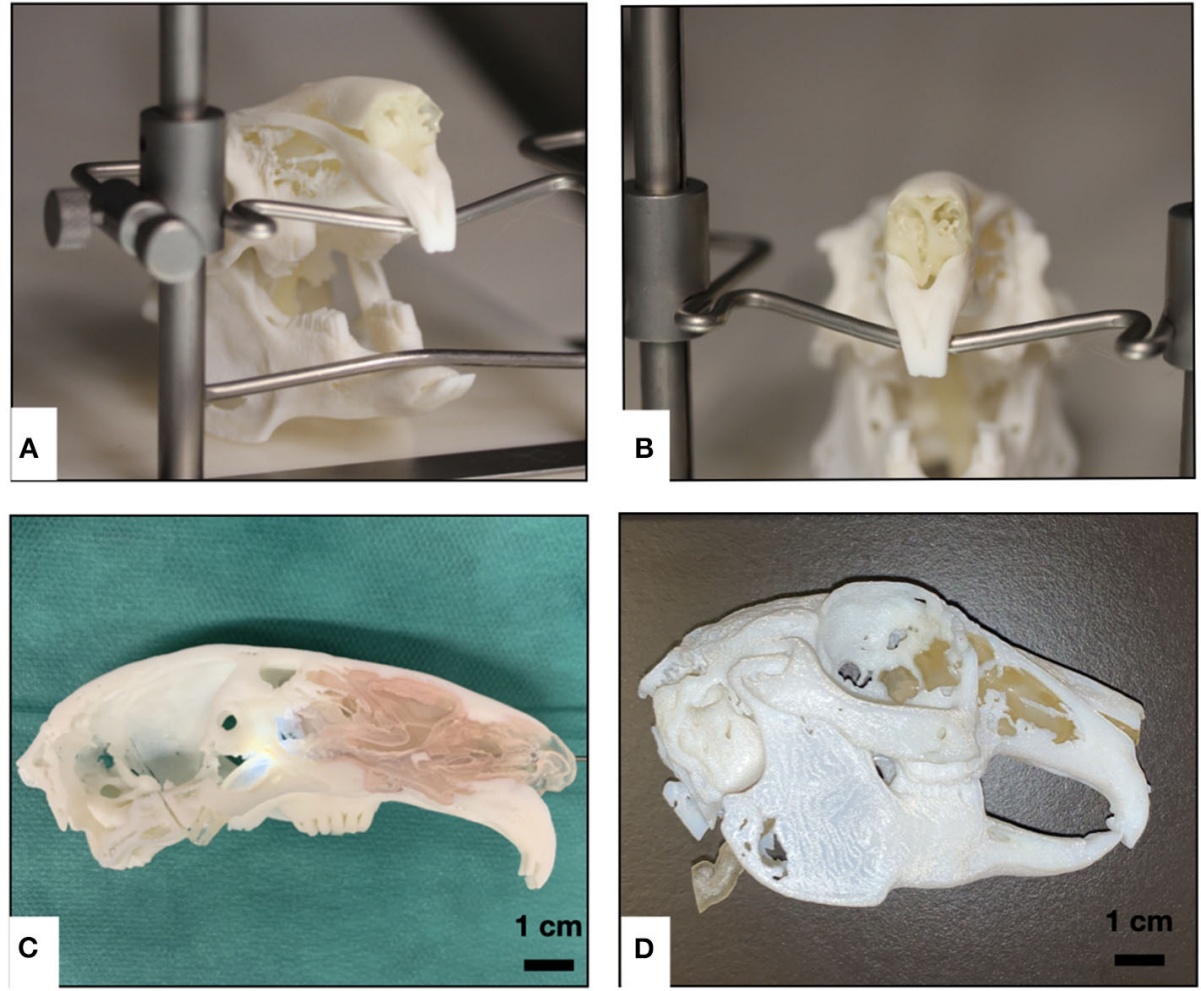
Overview of different multi-material 3D printed rabbit airway models using polyjet technology. **(A)** Intact μCT-based model rostro-lateral view, **(B)** μCT-based model rostral view of the nasal soft tissues, **(C)** μCT-based sagittal cross-sectional model view with pink-colored soft tissue, and **(D)** intact CBCT-based model lateral view.

The anatomical landmarks on the STL (digital) models were accurately replicated on the 3D printed models with a printing resolution of 600 dpi (42.3 μm) in the XY plane and a layer thickness of 30 μm in the Z direction (Figure 6).

### Dimensional Accuracy

**Figure 7:**
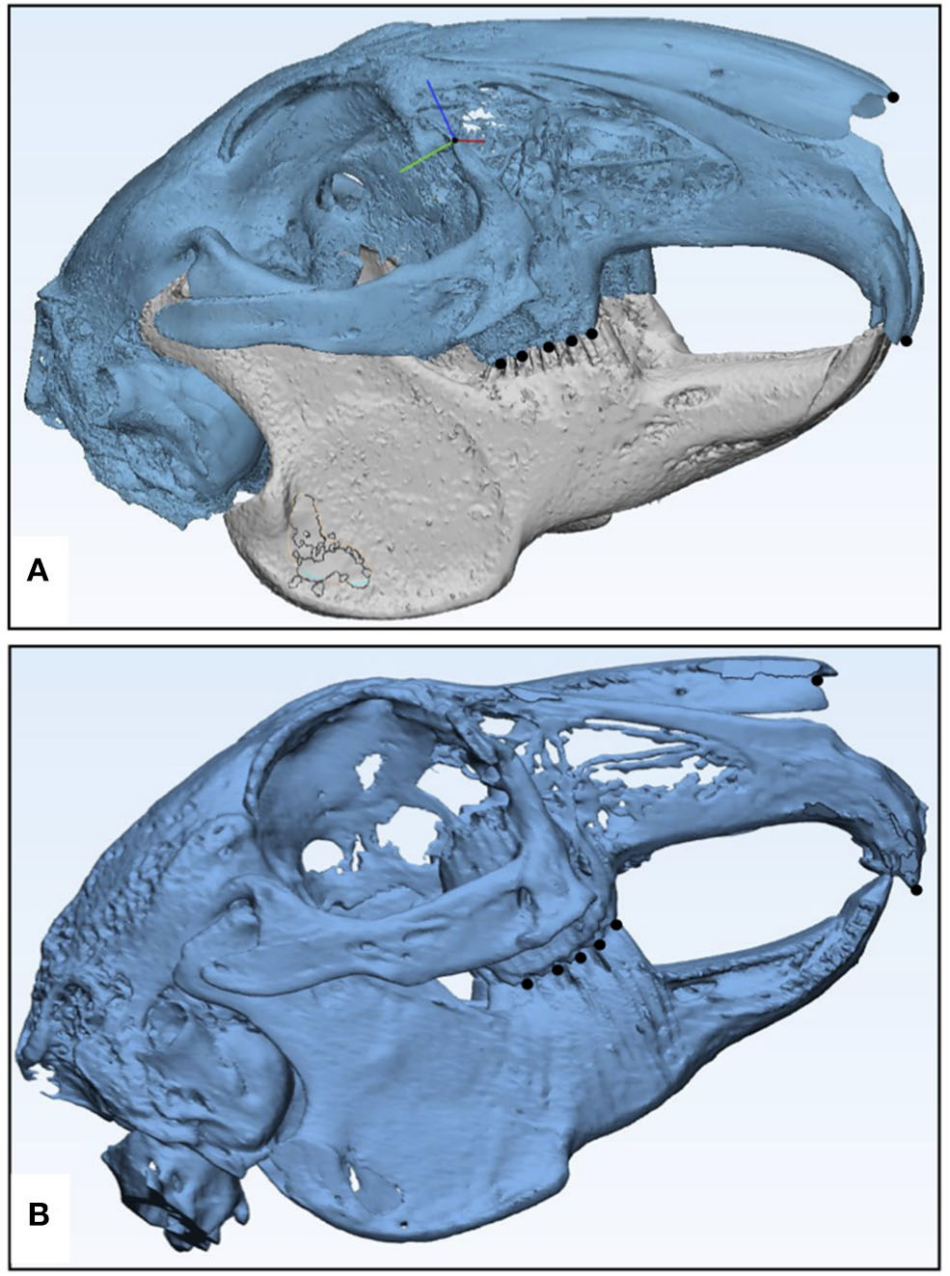
Digital model or STL files of μCT-based rabbit airway models created in Materialize 3-Matic 13.0 software for dimensional accuracy comparison. The black dots represent the anatomical landmarks chosen for the registration of specimen STL on model STL. **(A)** μCT-derived model STL and **(B)** CBCT-derived model STL.

**Figure 8:**
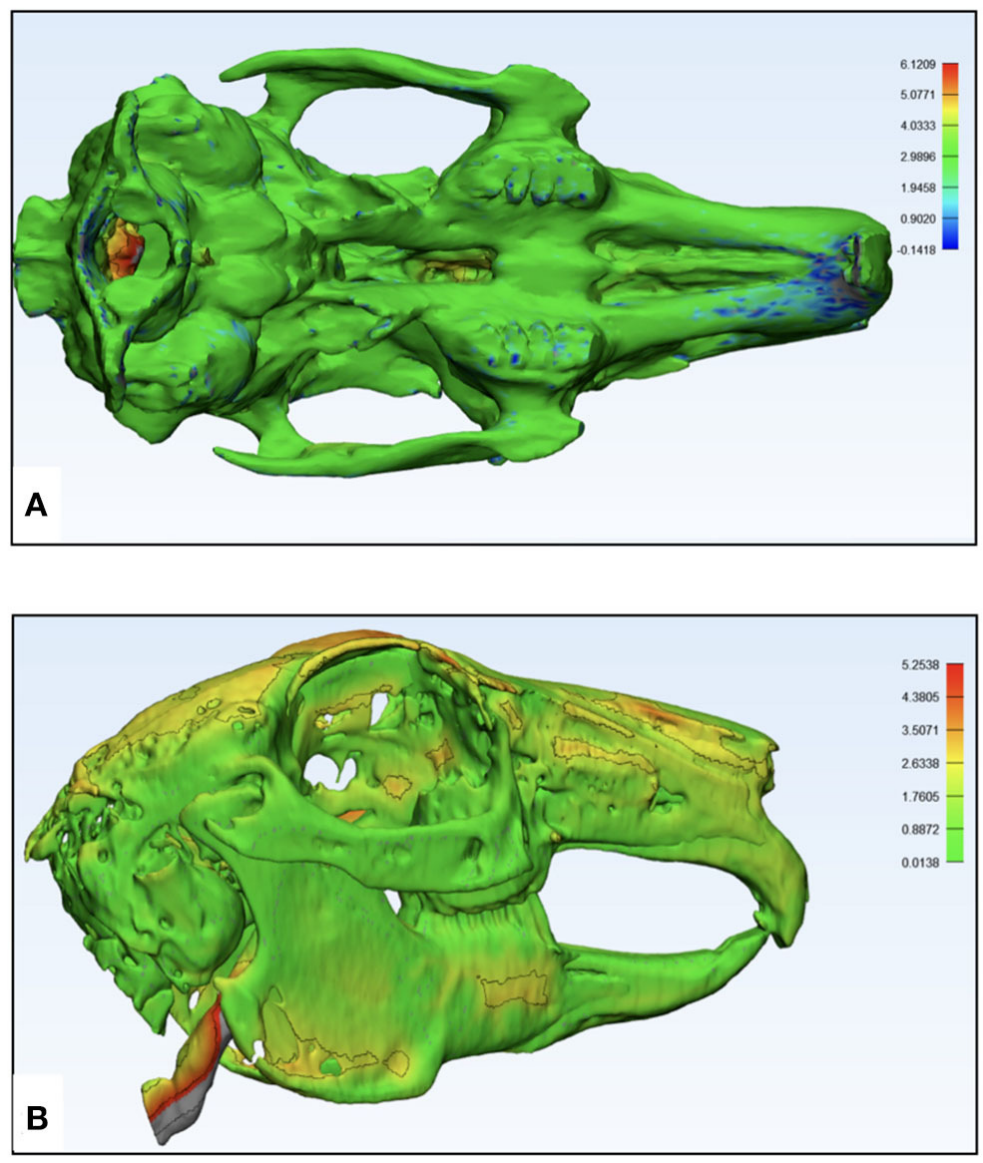
Pictorial representation of the dimensional accuracy of the additively manufactured rabbit airway models using part comparison analysis in Materialize 3-Matic 13.0 software. The color codes green, yellow, orange, and red signify the accuracy of the overlapping points, which is quantified by the side bars. **(A)** μCT-based model displaying an excellent overlap and a mismatch in the occipital part of the skull and **(B)** CBCT-based model displaying an excellent overlap and a minor surface mismatch.

**Figure 9:**
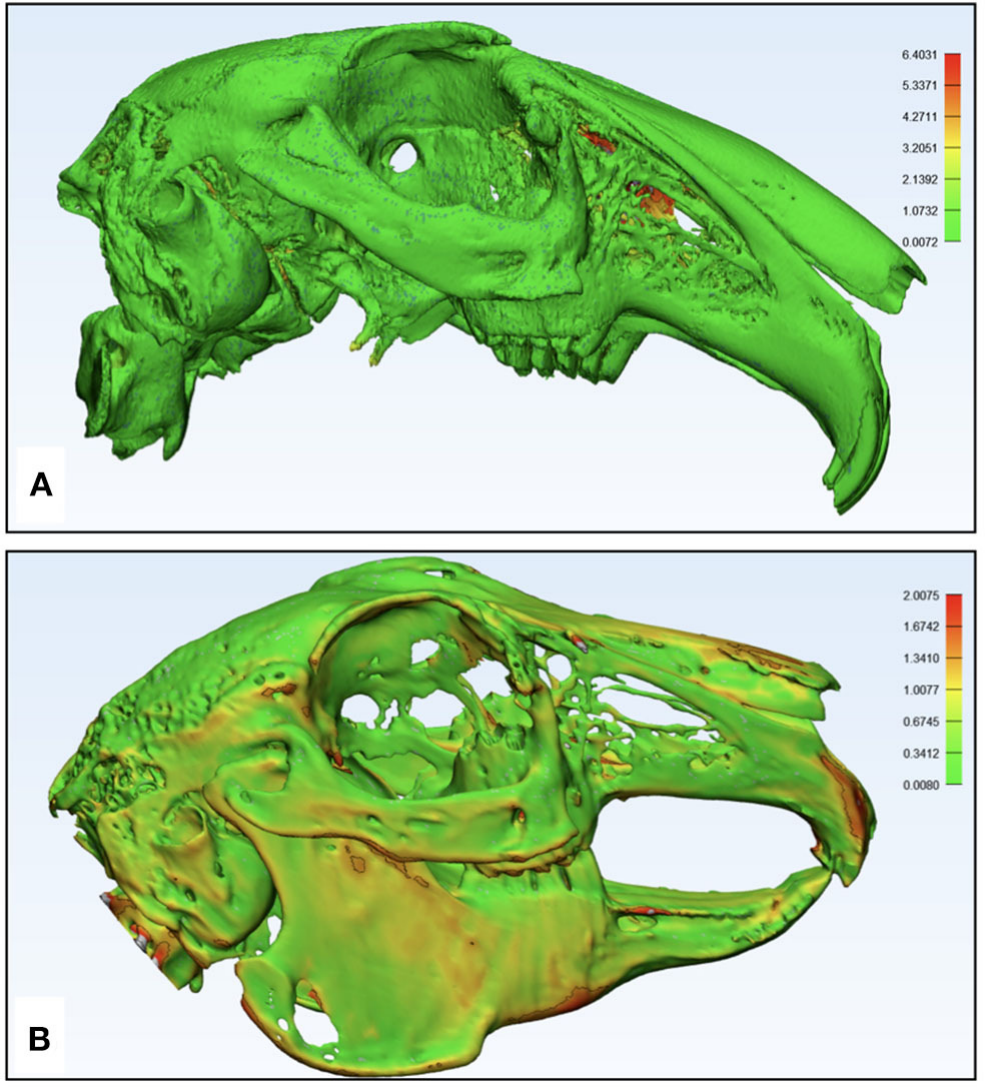
Pictorial representation of the imaging-based printing efficiency of the polyjet printer analysis of replication of specimen anatomy on the model using Materialize 3-Matic 13.0 software. The color codes green, yellow, orange, and red signify the anatomical structures on the specimen's skeleton accurately translated to the model, which is quantified by the side bars. **(A)** μCT-based STL of specimen 1 displaying the detailed nasal anatomy not printed in the model and **(B)** CBCT-based STL of specimen 2 displaying most of the skeletal parts that were translated into the model.

The 3D printed models of both specimens were rescanned to generate STL files for evaluation of dimensional accuracy (Figure 7). Only the skeletal component on the STLs of the models and original specimens was compared. From overall volumetric measurements, it was found that the total volume of μCT-derived model STL was 20,000.14 mm^3^ and that of CBCT-derived model STL was 19,000.32 mm^3^, while the volumes of animal specimens 1 and 2 were 21,000.67 and 19,362.64 32 mm^3^, respectively. The initial set of results from the part comparison analysis showed that 85% of the surface entities (also referred to as particles/points) had an error below 2.5 mm (Table 1). The segmentation analysis of the parts showing maximum surface entity mismatch in the range of 7.6–10.0 mm was a result of residual support material in the hollow parts of the skull (Figure 8). The models underwent a second cleaning cycle to remove the support material. The second part comparison analysis showed that 93% of the points present on the model 1 and 97% of the points on model 2 were dimensionally accurate representations of their respective animal specimen STLs within a threshold range of 0.0–2.5 mm (Table 1). From our analysis, we also found that only 84% of the points on specimen 1 μCT STL was accurately reproduced in the additively manufactured model 1, while the number was much higher, up to 95%, in the CBCT-derived model (Figure 9, Table 2). The range of error was higher in the μCT-derived model as compared to that in the CBCT-derived model (Table 2) and was mainly seen in the complex skeletal anatomy in the nasal and the paranasal areas.

**Table 1:** Results from the dimensional accuracy evaluation of the additively manufactured rabbit airway models using part comparison canalysis in Materialize 3-Matic 13.0 software before and after the removal of support material.

**Threshold (mm)**	**% of overlapping particles**		
	**μCT model**	**μCT model**	**CBCT model**
	**With support material %**	**Without support material %**	**Without support material %**
I (0.0–2.5)	85.00	93.00	97.00
II (2.6–5.0)	9.00	4.00	1.00
III (5.1–7.5)	2.00	2.00	1.00
IV (7.6–10.0)	4.00	1.00	1.00

*The color coding is in synchronization with the pictorial representation in Figure 8*.

**Table 2:** Results of the quantitative analysis of the imaging-based printing efficiency of the polyjet printer.

**Threshold (mm)**	**μCT specimen**	**Threshold (mm)**	**CBCT specimen**
	**% of points replicated on model 1**		**% of points replicated on model 2**
I (0.0–2.5)	84.00	I (0.0–1.2)	95.00
II (2.6–5.0)	13.00	II (1.2–2.5)	5.00
III (5.1–7.5)	2.00	III (2.5–3.7)	0.00
IV (7.6–10.0)	0.00	IV (3.7–5.0)	0.00

*Comparison of the replication of skeletal entities on specimens 1 and 2 onto models 1 and 2, respectively. The color codes green, yellow, orange, and red signify the accuracy of the overlapping points, which is quantified by the side bars as defined in the Figures 8, 9*.

### Endoscopic Evaluation

**Figure 10:**
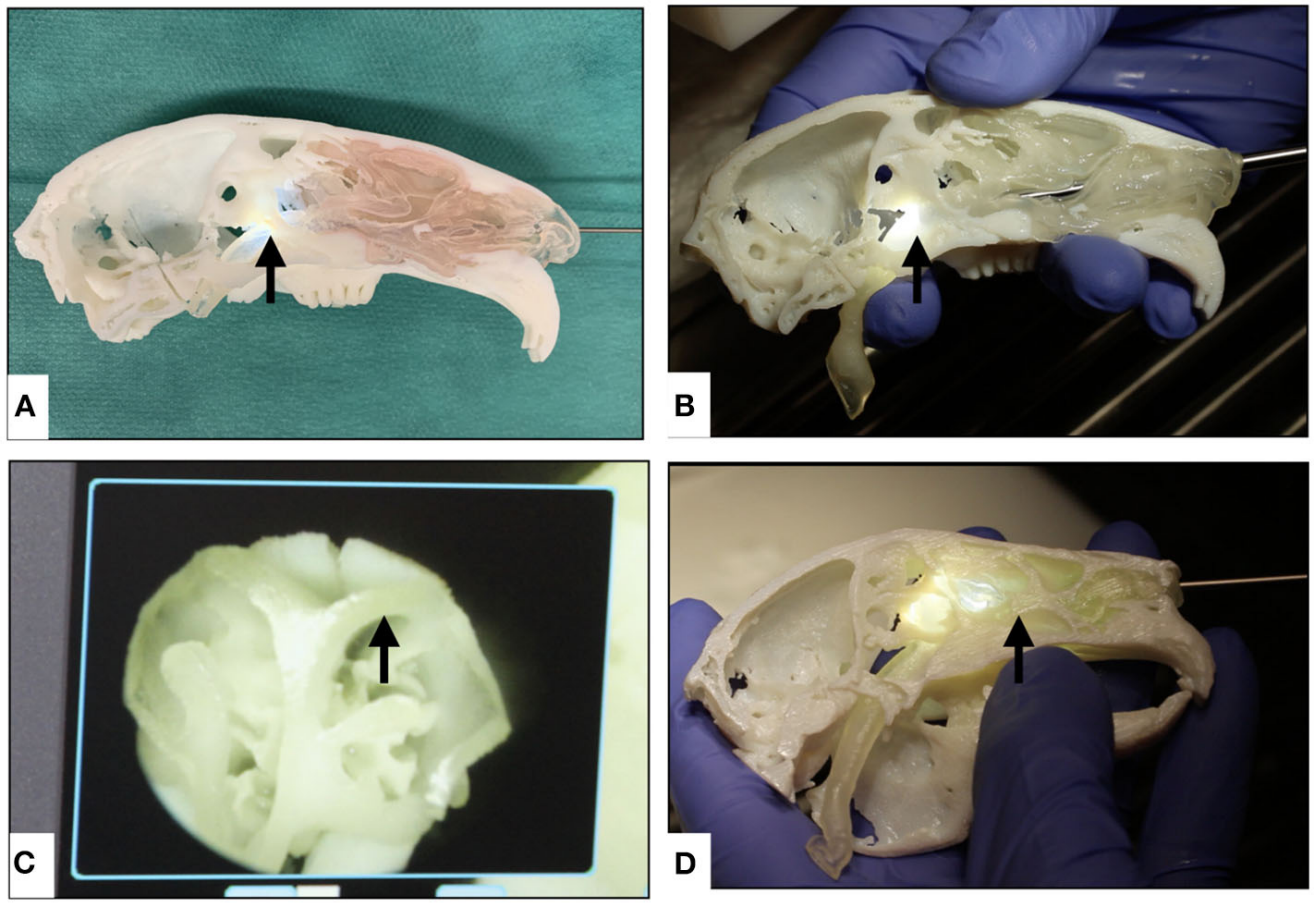
Overview of nasopharyngeal endoscopy in additively manufactured rabbit airway models using 1- and 2-mm rigid endoscopes. **(A)** Nasopharyngeal endoscopy in μCT-based model using a 1-mm endoscope, **(B)** nasopharyngeal endoscopy in μCT-based model using a 2-mm endoscope, **(C)** endoscopic view of the nasal cartilage in the model, **(D)** nasal endoscopy in CBCT-based model using a 1-mm endoscope showing that the endoscope could not go through the choana (the black arrows indicate the distal-most tip of the endoscope in each model).

**Figure 11:**
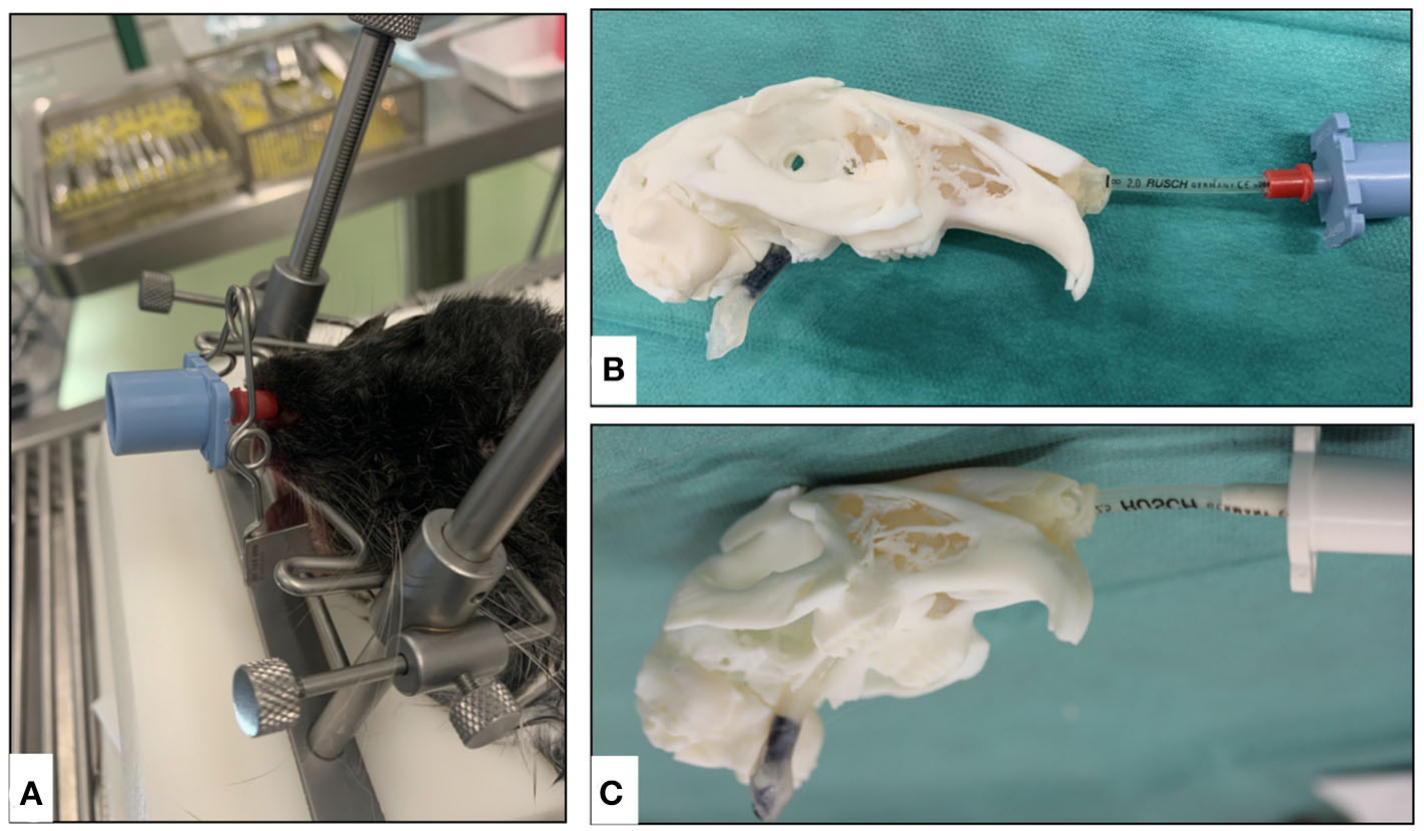
Overview of the nasotracheal intubation in animal specimen and additively manufactured models. **(A)** Animal specimen intubation using a 2-mm tube, **(B)** model 1 intubation using a 2-mm tube, and **(C)** model 1 intubation using a 2.5-mm tube.

**Figure 12:**
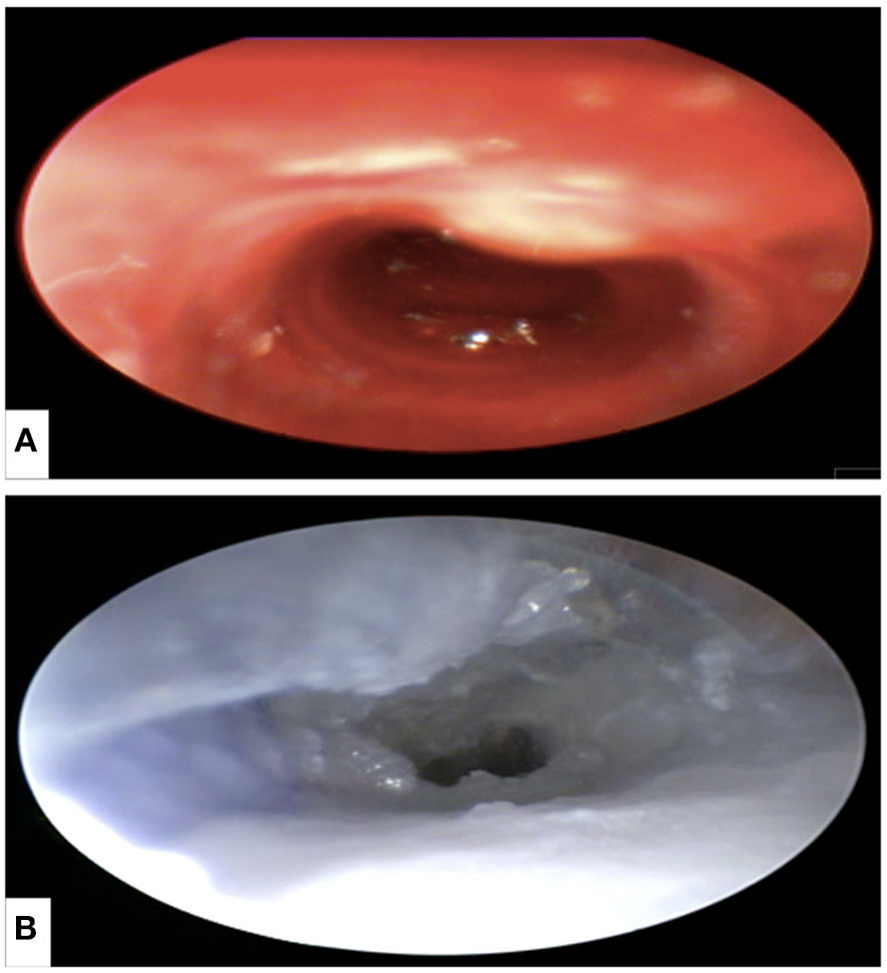
Nasotracheal endoscopy from the larynx showing the first two tracheal rings of the trachea. **(A)** Animal specimen and **(B)** μCT-based model.

It was possible to perform nasal endoscopy (from the nasal plane to the nasal canthus of the eye) using a 1-mm rigid endoscope in both model 1 and with limited depth in model 2 (Figure 10), while using a 2-mm rigid endoscope was only possible in model 1. Nasotracheal intubation using 2 and 2.5-mm tubes was only possible in model 1 (Figure 11). The endoscopic view of model 1 was comparable to the real anatomic view of the animal (Figure 12). During endoscopy, the tactile sense of inserting and walking through the synthetic nasal cavity from the resistant point of view was nearly the same as in living rabbits.

### Digital Microscopy Confirmed the Absence of Support Material on the Model Surface

**Figure 13:**
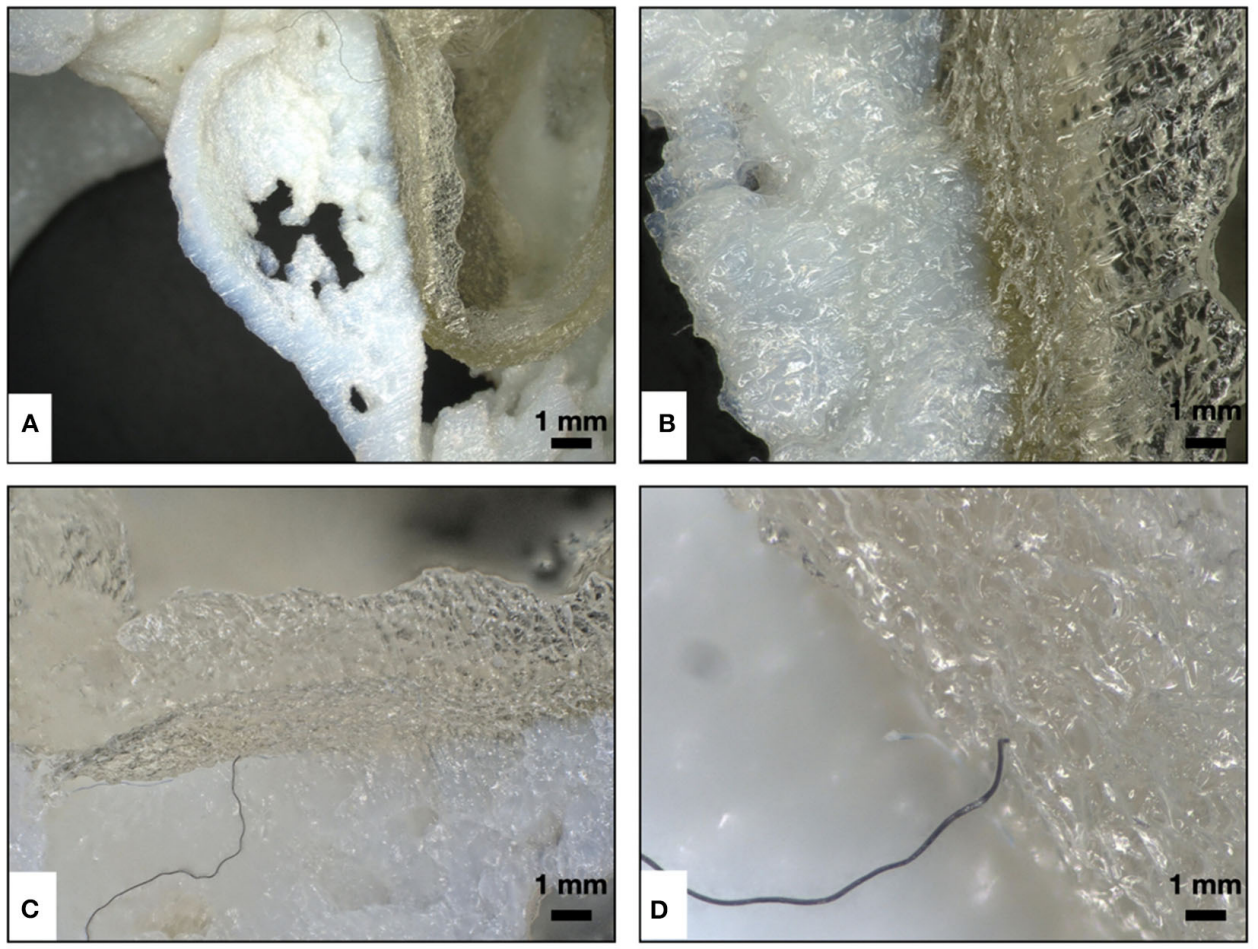
Qualitative assessment of surface cleaning of the model signifying the removal of support material (SUP 706) using digital microscopy. **(A,B)** Image under ×20 magnification, showing both the hard and the soft model surfaces that were free of support material. **(C,D)** Image under ×20 magnification, showing the inclusion of a fiber in the printing material during the printing process.

By carefully examining the surface of both models under ×20 magnification, it was seen that the model surfaces were free of support material (Figure 13). However, we found inclusion of a fiber into the printing material which could have been incorporated during the printing process (Figures 13C,D).

### Radiation Attenuation Evaluation

According to Figure 4, we observed that, despite limitations in contrast of the bony parts, the difference between bony and mucosal areas is visually detectable. In addition, the trachea on both models is also visible. The resulting HUs are presented in Table 3. The results of this analysis show 234.46 and 259.05 HU differences in their mean radiodensities between the mucosal and the bony areas for models 1 and 2, respectively. Our results based on both visual inspection and quantitative analysis (by HU analysis) show the distinguishability between the mucosal and the bony areas. The high standard deviation observed in the mucosal area could be attributed to the structural heterogeneity of the region of interest (air, nasal cartilage, and mucosa).

**Table 3:** Results of quantitative radiodensity analysis using Analyze 12.0 tool kit.

**Parameters**	**μCT model**		**CBCT model**	
	**Skeletal part**	**Mucosal part**	**Skeletal part**	**Mucosal part**
Maximum	301	107	312	116
Minimum	277	−27	289	−80
Mean	290.08	55.62	300.17	41.12
Standard deviation	6.96	45.62	9.56	49.37

*The table shows the minimum, maximum, mean, and standard deviation of the radiodensities in the region of interest in skeletal and mucosal parts in μCT- and CBCT-derived models*.

## Discussion

Nasotracheal intubation exploits the property that rabbits are obligate nasal breather. Rabbits typically have their epiglottis attached to the dorsal surface of the oropharynx, in this manner permitting the direct entry of air from the nasopharynx into the larynx and the trachea. A catheter/tube inserted nasally will normally cross this pathway from the nasopharynx into the larynx and the trachea (37). In spite of the fact that nasotracheal intubation has been depicted quickly as an elective strategy for intubation in rabbits, restricted literature on this method is accessible, and contraindications incorporate the chance of bringing pathogens into the lungs and the requirement for high oxygen stream rates (38). One study shows that rabbits that received nasotracheal intubation were followed up for more than 2 months, and no clinical indications of respiratory infection were noted (38). Moreover, high oxygen stream rates were superfluous. This report depicted effective nasotracheal intubation of rabbits and a few key emphases for suitable clinical utilization of nasotracheal intubation. It has been proven that nasotracheal intubation gives negligible injury, dissimilar to recently revealed laryngeal wounds from orotracheal intubation (38, 39).

To gain efficient tissue handling, past attempts have been made to reproduce anatomical animal models using fused deposition modeling as an adjunct to veterinary clinical training (40–42). With the introduction of 3D printed animal models, providing superior vision of anatomy as compared to the two-dimensional pictorial representations, it is possible to visualize the complex anatomical arrangement in advance of animal surgery (43–47). The accuracy of our medical imaging-based models produced with polyjet technology and the reproducibility of hard and soft tissue anatomy is of great value to institutions for adopting an additive manufacturing-based educational system (43). The material selection representing the anatomical components was inspired from previous works where Vero Pure White and Tango Plus has been used to represent bone in other 3D–printed medical applications such as the thorax and have been used to substitute hard and soft tissues in models (35, 48, 49). This adds to the tactile experience of the trainees.

The most indispensable step of 3D printing technology is cross-sectional image acquisition and segmentation, where radiology plays an elemental role (45). Our study suggested that μCT and CBCT are efficient tools for visualizing the micro- and macro-anatomy, respectively (43), and μCT gave more detailed information about the labyrinthine nasal and paranasal anatomy. However, only 85% of this information was precisely (in the range of 0.0–2.5 mm) translated into the model due to limitations in the contrast-based segmentation process. This had an impact during nasal endoscopy and intubation, as the models derived from CBCT could not be investigated through the full length of the nasotracheal pathway, while the μCT-derived models were intubated with up to 2 mm tubes through the trachea. In our workflow, we have also shown that CT scanning of the AM models in polymers offers the possibility of dimensional measurements. CT scan is a beneficial tool to analyze the quality and the precision of AM models. This technology provides an advantage over other mechanical/optical measurement tools, as it also provides a detailed cross-sectional view of the internal structures, including the surface. The high-resolution CT imaging is hence an additional measurement system for quality management and continuous proof. As an adjunct to CT, digital microscopy in ×20 magnification can be advantageous in checking the surface quality of the 3D printed models and confirm the absence of support material.

The dimensional accuracy of ~93 and 97% in μCT- and CBCT-derived models, respectively, after appropriate cleaning and life-like representation of these models also helps to develop medical devices, surgical cutting guides, drills, and implants specifically customized for the smaller anatomical structures of these animals and to avoid iatrogenic trauma during routine clinical procedures. Injuries to the larynx and the vocal cords of rabbits during endotracheal intubation often results in incorrigible damage, and hence to avoid such loss, these models prove to be a boon. With advancement in infrastructure and material science, we can incorporate blood vessels, body fluids, and critical nerves inside the model, so the clinician/surgeon can self-monitor the level of trauma caused by instrumentation. This model is advantageous for the selection of the correct size of diagnostic and surgical instruments for prospective dissection approaches. With increasing competence of mimicking the physical properties of the musculoskeletal structures in the computer-generated 3D printed models, it provides a more efficient proprioception to the surgeon or engineer (50, 51). Thus, our bio-mimetic model is a promising approach to bridge the gap between science and ethics, greatly reducing animal dependency.

In this study, we have evaluated the whole workflow, from image acquisition to the reconstruction of a 3D animal model in an acceptable quality for multiple research purposes. Optimal model cleaning appeared to be an important step to avoid support material encapsulation and open big holes for the cleaning. With the detailed segmentation of part comparison analysis, it was possible to see the presence of support material in the occipital part of the skull. The analysis shows that the high-dimensional mismatch (2.2–8.7 mm) was based on the residual support material which was used during the printing process (SUP 706). It was not possible to clean the model completely from the support material due to the fragility of the intricate nasal structures. Additionally, the support material was encapsulated within the cancellous geometry of the bone. This analysis combination of CT scan and Materialize 3-Matic 13.0 software (Materialize, Leuven, Belgium) can be applied in quality management to discern the cleaning efficiency of the post-printing support material removal process of the model as higher failure rates (>0.5 mm) can be caused due to the residual support material. During nasal endoscopy, it was often required to flush the remnant support material to improve the patency of the airway. Newer systems based on polyjet technology use a water-soluble support material (SUP 707) which would be easier to remove.

Along with geometrical precision and anatomical replication, our models also offered limited radiographic information. The radiodensities of the skeletal and the mucosal parts of the models displayed a difference of 234 and 259 HU in μCT- and CBCT-derived models, respectively. This can be utilized in gaining hands-on experience in imaging-based diagnostic and therapeutic treatment procedures. However, this difference was not optimal enough to quantify the anatomical discrepancy between the original STL and the model STL. A further limitation of our study was its inability to obtain a realistically colored material which could have added an advantage in endoscopic experience. Our research was a pilot study, lacking a follow-up of the participants' endoscopic practice. The weakest feature of the model was the difference in realism of nasal cartilage and mucosa due to the elastic consistency of the Tango Plus material. With the availability of bioprinting technology, organs and tissues can be directly printed with a combination of bioinks that provide the possibility to replicate the biomechanical properties of tissues more accurately (52, 53). However, the aim of the current study was to develop models that can be repeatedly used to train nasotracheal intubation for a longer duration, which hence could be a concern with bioprinted models. Further research would be aimed at developing a 3D printed model with materials having more representative colors, biomechanical properties, and radiation attenuation in the soft and hard tissue regions of the head, making it a useful tool for skills training.

In conclusion, our research shows that μCT is undoubtedly a superior technology to fabricate musculoskeletal simulation models; however, only 84% of the complex structural entities consisting of heterogenous soft tissues smaller than 2.5 mm can be accurately translated onto the 3D printed model using polyjet technology. This indicates that a further improvement in segmentation technique and computer-aided designing software is required. With enhancement in the radiodensity properties of the hard and soft printing materials, it would be possible to increase the radiation attenuation properties of the simulation models and contribute to the field of imaging phantoms. Simultaneously, the appropriate removal of support material is imperative for improving model accuracy and avoiding false negative results. Additionally, our workflow shows the possibility to include medical imaging and digital microscopic technologies inside a quality management program for the quantitative and qualitative analyses of additively manufactured polymer models.

## Data Availability Statement

The original contributions presented in the study are included in the article/supplementary materials, further inquiries can be directed to the corresponding author/s.

## Ethics Statement

Ethics permission was not required for our study since we used sacrificed animals from an ongoing study in the Department of Biomedical Research, Medical University of Vienna, Austria and the Department/Hospital for Companion Animals and Horses, University of Veterinary Medicine, Vienna, Austria. The ethical permission for their ongoing studies was already acquired by the Ethic Commission of the University of Vienna.

## Author Contributions

GO was involved in study design, experimental work, literature research, data analysis, writing the manuscript, and submission process. ME-S, A-MK, and DB were involved in animal experiments, imaging, and writing. SH and PS were involved in imaging and data analysis. MK was involved in 3D printing. HA, FM, and EU were involved in study design, writing, and submission process. All authors contributed to the article and approved the submitted version.

## Conflict of Interest

The authors declare that the research was conducted in the absence of any commercial or financial relationships that could be construed as a potential conflict of interest.
